# A Cyclopedia of Practical Medicine and Surgery

**Published:** 1900-11

**Authors:** 


A Cyclopedia of Practical Medicine and Surgery. A concise reference book, alphabetically arranged, of Medicine, Surgery, Obstetrics, Materia Medica, Therapeutics, and the various specialities, with particular reference to Diagnosis and Treatment. Compiled under the editorial supervision of George M. Gould, A. M., M. D., editor of the *"Philadelphia Medical Journal,"* etc., and Walter L. Pyle, A. M., M. D., Assistant Surgeon to Wills Eye Hospital. Seventy-three contributors. Quarto, illustrated. Sheep or half dark green leather, $10.00; thumb index, $11.00; half Russia, thumb index, $12.00. P. Blakiston's Son & Co., Philadelphia. 1900.

To those who are familiar with Quain's Dictionary of Medicine this book will be appreciated. It has no index, but the subject matter is arranged in alphabetical order, with bold-face headings, so one can easily turn to any subject. It covers succinctly the whole domain of medicine and surgery, and should be invaluable to busy physicians and students. The chief feature is the diagnosis and treatment, but each subject is so elaborated that a comprehensive view is readily had. The book has no equal of the kind in this country.

